# The role of causal reasoning and directed acyclic graphs in multivariable regression: implications for evidence-based veterinary medicine

**DOI:** 10.3389/fvets.2026.1774492

**Published:** 2026-03-18

**Authors:** Pablo A. Donati, Felipe J. Lillo-Araya, Marina Valdora, Pablo E. Otero

**Affiliations:** 1 Department of Anesthesiology and Pain Management, Facultad de Ciencias Veterinarias, Universidad de Buenos Aires, Buenos Aires, Argentina; 2 Facultad Ciencias de la Vida, Escuela de Medicina Veterinaria, Universidad Andres Bello, Santiago, Chile; 3 Facultad de Ciencias Exactas y Naturales, Institute of Calculus, Universidad de Buenos Aires, Buenos Aires, Argentina

**Keywords:** causal inference, confounding control, directed acyclic graphs, multivariable regression, observational studies

## 1 Introduction

The term evidence-based medicine, coined by Dr. Guyatt in 1991 (1), describes the practice of medicine rooted in the best available scientific evidence (2). Since its inception, evidence-based medicine has significantly transformed the global application of scientific knowledge in clinical practice. From an epistemological perspective, it is founded on the idea that the justification of a belief depends on the degree and quality of supporting evidence. Accordingly, a crucial tenet of evidence-based medicine is the systematic evaluation of all available evidence rather than ignoring evidence that contradicts preferred beliefs (2, 3).

The strength and relevance of evidence depend critically on the type of research question being addressed and the appropriateness of the corresponding study design. This relationship is traditionally illustrated in the traditional evidence pyramid, where studies that offer the lowest level of evidence are placed at the bottom, while those that provide the highest level are at the top (4). However, this hierarchy was largely developed for interventional questions, where random allocation is feasible, and may be less informative for etiological or prognostic research, which are highly relevant in veterinary medicine.

Randomized controlled clinical trials are generally considered the strongest form of evidence derived from individual original research. Nevertheless, even well-conducted trials may suffer from limited internal validity, that is, the extent to which the design, conduct, and analysis of a study allow for an unbiased estimate of the causal effect within the study population, free from systematic error such as confounding, selection bias, or measurement bias. Their prominence in evidence hierarchies should not be automatically generalized to all research questions (2, 4). In veterinary medicine, however, many clinically relevant questions are not primarily interventional but etiological in nature, aiming to identify causes, risk factors, and mechanisms of disease.

Randomized controlled clinical trials are founded on counterfactual theory, which provides a framework for answering important research questions (5). Because the investigator controls the exposure, randomization is possible in this setting (6). In contrast, etiological questions typically involve exposures that cannot be experimentally assigned, making observational studies the preferred, and often the only feasible, design.

A confounder is a variable that is a common cause of both the exposure and the outcome and is not on the causal pathway between them (7). In controlled clinical trials, if the sample size is adequate, potential known and unknown confounders are expected to be evenly distributed between comparison groups (5, 6). In observational research, however, comparison groups may differ systematically, potentially threatening causal inference. Although observational studies can yield valuable scientific insights—particularly for etiological and prognostic questions when randomization is not feasible or ethically justifiable (8)— the absence of randomization means that it becomes necessary to address potential confounding through design-based strategies (such as restriction or matching) and analytical approaches (such as statistical adjustment).

Because observational studies lack random allocation, valid causal inference depends not only on statistical adjustment but also on appropriate design-based strategies and, more fundamentally, on explicit consideration of the underlying causal structure linking exposures, outcomes, and covariates. Distinguishing between confounders, mediators, and collider variables is therefore essential for avoiding biased effect estimates. In this context, the aim of this Opinion article is to examine the role of observational studies within evidence-based veterinary medicine, with particular emphasis on the correct identification and control of confounding. We focus on the conceptual distinction between confounders and mediators, as well as collider variables, discuss the limitations of multivariable regression models when causal assumptions are not explicitly articulated, and argue that directed acyclic graphs (DAGs) provide a rigorous and clinically intuitive framework for improving causal inference in veterinary observational research.

## 2 Directed acyclic graphs and causal structure in observational research

### 2.1 Conceptual foundations of directed acyclic graphs

Directed acyclic graphs (DAGs) are graphical representations of hypothesized causal relationships among variables in a given research question. In a DAG, each variable is represented as a node, and arrows denote the assumed direction of causal influence between variables. The term *directed* refers to the presence of arrows with a defined orientation, whereas *acyclic* indicates that feedback loops are not allowed; no variable can be both a direct or indirect cause of itself.

Nodes may represent patient characteristics, exposures, physiological processes, treatments, or outcomes, and are defined based on clinical and biological knowledge rather than statistical associations. Arrows encode causal assumptions, such that an arrow from variable A to variable B indicates that A is believed to causally influence B. Importantly, the absence of an arrow is also informative, as it reflects the assumption that no direct causal relationship exists between two variables.

Within this framework, DAGs allow for a clear conceptual distinction between confounders, mediators, and colliders, based on their position in the causal structure rather than on their statistical behavior. A confounder is a variable that causally affects both the exposure and the outcome, thereby generating a non-causal association between them. Adjustment for confounders is necessary to block these so-called backdoor paths and to obtain unbiased estimates of causal effects (7).

A mediator lies on the causal pathway between the exposure and the outcome and represents a mechanism through which the exposure exerts its effect. Adjusting for a mediator blocks part or all of the causal effect of interest and therefore leads to biased estimation of the total effect (7). Despite this, mediators are frequently misclassified as confounders in veterinary observational research, particularly when they are physiological variables measured after exposure.

A collider is a variable that is causally influenced by two or more other variables, a structure represented in a DAG by two arrows pointing into the same node. Conditioning on a collider, either through stratification or regression adjustment, opens a spurious association between its causes, thereby introducing selection bias. Although colliders may appear statistically associated with both exposure and outcome, this association is non-causal and arises solely from conditioning on a common effect. Failure to recognize colliders is a frequent source of bias in observational studies, particularly when post-exposure variables or markers of disease severity are inappropriately adjusted for.

DAGs also provide a transparent method for identifying backdoor paths, which are non-causal pathways connecting exposure and outcome through common causes. A valid adjustment set is one that blocks all backdoor paths without conditioning on mediators or colliders. This principle underscores why variable selection cannot rely exclusively on statistical criteria and must instead be guided by explicit causal reasoning.

By making causal assumptions explicit, DAGs do not replace statistical models but guide their construction. They ensure that multivariable regression models are aligned with the causal question of interest, thereby improving interpretability and internal validity. In veterinary observational research, where exposures are rarely randomized and complex physiological processes are often involved, DAGs offer a clinically intuitive and methodologically rigorous framework for causal inference.

**Figure 1:**
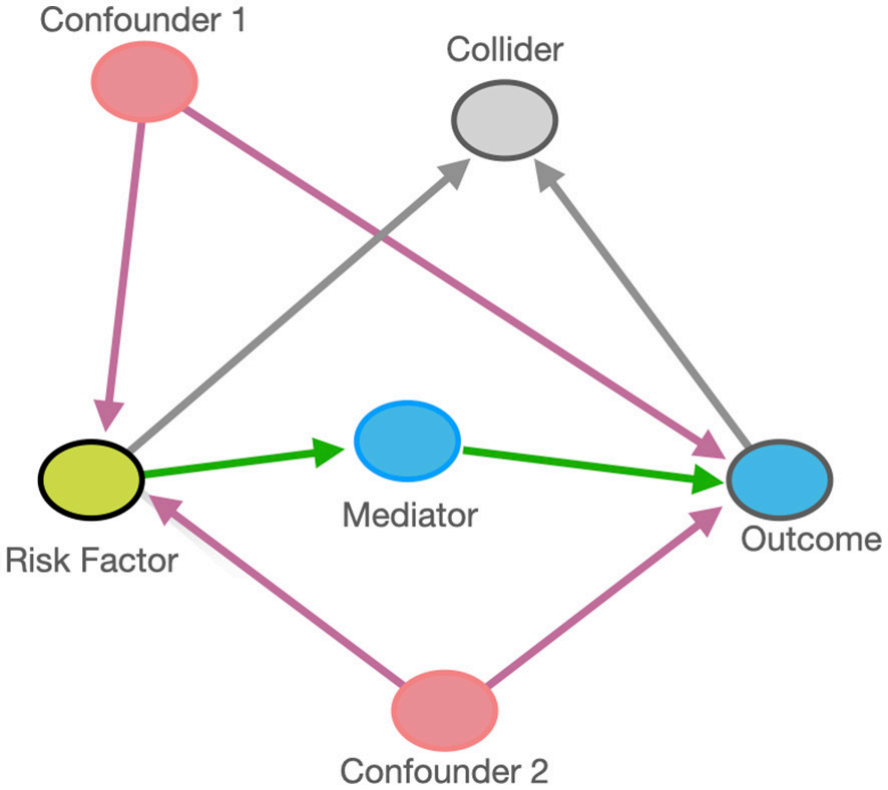
Directed acyclic graph (DAG) illustrating the relationships among a risk factor and an outcome, including confounders, a mediator, and a collider. Confounders (Confounder 1 and Confounder 2) are common causes of both the risk factor and the outcome. The mediator lies on the causal pathway between the risk factor and the outcome. The collider is a variable influenced by two other variables and should not be conditioned on, as doing so may induce spurious associations. Arrows represent assumed causal directions.

Figure 1 presents a simplified, hypothetical DAG illustrating the relationships between an exposure, an outcome, and common sources of bias (confounding, mediation, and collider structures) in observational research.

### 2.2 Confounding control and regression models

To preserve internal validity, researchers must attempt to identify and appropriately control for confounding variables (7, 8). Common strategies for confounding control in observational studies include stratification, standardization, and regression modeling (8). Regression models are particularly attractive because they allow simultaneous adjustment for multiple confounders.

Depending on the nature of the outcome variable, different regression models are used, including linear regression for continuous outcomes, logistic regression for binary outcomes, Poisson regression for count data, and proportional hazards (Cox) regression for time-to-event outcomes (8). It is important to distinguish between multivariate and multivariable regression, terms that are often used interchangeably but describe different analytical approaches (9). Multivariate regression involves multiple outcome variables, whereas multivariable regression includes a single outcome with multiple independent variables (10).

While multivariable regression can reduce bias due to measured confounding, it cannot account for unmeasured or unknown confounders (6). Furthermore, randomization promotes exchangeability between comparison groups in expectation, meaning that, on average, measured and unmeasured confounders are balanced across arms. However, in any given trial, especially those with limited sample size, some imbalance may still occur by chance. Only in sufficiently large trials does randomization tend to achieve approximate balance due to the law of large numbers. In contrast, regression adjustment in observational studies relies entirely on measured variables and modeling assumptions. Therefore, regression adjustment should not be interpreted as establishing causality, but rather as an attempt to approximate causal effects under explicit assumptions.

A critical limitation of regression modeling is that statistical associations alone cannot distinguish confounders from other types of variables within the causal structure. In particular, mediators, variables that lie on the causal pathway between exposure and outcome, are frequently misclassified as confounders and adjusted for inappropriately. Conditioning on mediators blocks part of the causal effect of interest and may lead to biased or misleading estimates (7).

Automated variable selection procedures, such as stepwise selection, are widely implemented in statistical software (11). These methods may rely on forward selection, backward elimination, or combinations of both (12). However, purely data-driven approaches risk including inappropriate variables, particularly mediators, thereby introducing bias (7, 13). More broadly, such approaches prioritize statistical criteria over causal reasoning and may inadvertently introduce collider bias when post-exposure variables are conditioned upon (13).

It is important to note that not all observational studies are designed to answer causal questions. Regression models are also widely used for prediction, where the primary objective is to estimate an outcome rather than to accurately identify causal effects. In predictive modeling, variables are selected based on their contribution to model performance, and causal concepts such as confounding, mediation, or collider bias are not directly applicable. The considerations discussed in this article specifically pertain to observational studies aimed at causal inference.

### 2.3 Illustrative veterinary applications of DAGs

The utility of DAGs has been demonstrated in recent veterinary studies. In a recent study on post-operative intestinal dehiscence in dogs, Donati et al. (14) used DAGs to clarify the relationship between preoperative septic peritonitis (PSP) and the risk of dehiscence. Their causal diagram identified hypoalbuminemia as a mediator rather than a confounder: PSP induces a systemic inflammatory response that lowers serum albumin, which may in turn impair wound healing and contribute to dehiscence. Because mediators lie on the causal pathway, adjusting for serum albumin would block part of the effect of PSP and produce a biased estimate of the total causal effect. In contrast, the DAG identified time from onset of clinical signs to surgery and the reason for surgery as true confounders, as both variables can influence the likelihood of developing PSP and independently affect the risk of dehiscence. Adjusting for these variables closes backdoor paths that would otherwise distort the estimated causal effect. This example illustrates how DAGs help researchers make principled decisions about which variables should, and should not, be included in regression models.

A second example comes from Portela et al. (15), who performed a retrospective non-inferiority study comparing the parasacral (PS) block with the recently developed greater ischiatic notch (GIN) plane block in dogs undergoing pelvic limb surgery. The GIN approach targets a fascial plane between the greater ischiatic notch and the piriformis muscle, thereby avoiding the parasacral space and potentially reducing complications such as nerve trauma or vascular puncture. Because block type was influenced by the complexity and duration of the orthopedic procedure, the DAG identified type of surgery and surgical time as confounders of the relationship between block type (exposure) and perioperative opioid consumption (outcome). In contrast, intraoperative hemodynamic interventions, post-operative NSAID use, or rescue analgesia occur downstream of the block or surgical stimulus and therefore function as mediators or colliders. The DAG-guided model correctly adjusted only for true confounders, preventing biased estimation of the block effect.

A similar application of DAGs can be found in critical care research. In the cohort study by Espiñeira et al. (16) comparing propofol vs. sodium thiopentone for the treatment of status epilepticus and refractory status epilepticus in dogs, the DAG explicitly illustrated how several pre-existing or concurrent clinical variables influence both treatment selection and patient outcomes. In their causal diagram, type of epilepsy was identified as a key pre-treatment factor affecting the choice of anesthetic agent. Metabolic and physiological disturbances, specifically hyperglycemia/hypoglycemia and hyperthermia, were depicted as influencing the risk of secondary brain injury, which in turn affects outcomes such as length of hospital stay and in-hospital mortality. Post-exposure variables, including hypotension and duration of therapeutic coma, were shown as consequences of the chosen anesthetic and contributors to downstream outcomes, functioning as mediators rather than confounders. By using a DAG, the authors clarified which variables must be treated as confounders (e.g., type of epilepsy) and which lie on the causal pathway and therefore should not be adjusted for when estimating the total effect of the anesthetic agent on patient prognosis.
